# Wettability and Surface Roughness of Parylene C on Three-Dimensional-Printed Photopolymers

**DOI:** 10.3390/ma15124159

**Published:** 2022-06-11

**Authors:** Fan-Chun Hsieh, Chien-Yao Huang, Yen-Pei Lu

**Affiliations:** 1 Department of Mechanical Engineering, National Chin-Yi University of Technology, Taichung 41170, Taiwan; 2 Taiwan Instrument Research Institute, National Applied Research Laboratories, Hsinchu 30076, Taiwan; msyz@narlabs.org.tw (C.-Y.H.); ypl@narlabs.org.tw (Y.-P.L.)

**Keywords:** Parylene C, 3D-printed, photopolymer, contact angle, surface roughness

## Abstract

The use of poly-(para-chloro-xylylene) (Parylene C) in microelectromechanical systems and medical devices has increased rapidly. However, little research has been conducted on the wettability and surface roughness of Parylene C after being soaked in solutions. In this study, the contact angle and surface roughness (arithmetic average of roughness) of Parylene C on three-dimensional (3D)-printed photopolymer in 10% sodium hydroxide, 10% ammonium hydroxide, and 100% phosphate-buffered saline (PBS) solutions were investigated using a commercial contact angle measurement system and laser confocal microscope, respectively. The collected data indicated that 10% ammonium hydroxide had no major effect on the contact angle of Parylene C on a substrate, with a Shore A hardness of 50. However, 10% sodium hydroxide, 10% ammonium hydroxide, and 100% PBS considerably affected the contact angle of Parylene C on a substrate with a Shore A hardness of 85. Substrates with Parylene C coating exhibited lower surface roughness than uncoated substrates. The substrates coated with Parylene C that were soaked in 10% ammonium hydroxide exhibited high surface roughness. The aforementioned results indicate that 3D-printed photopolymers coated with Parylene C can offer potential benefits when used in biocompatible devices.

## 1. Introduction

Polymers are attracting widespread interest for use in medical devices because of their high chemical resistance, biocompatibility, and optical transparency and low cost (lower than that of glass or silicon). Various polymeric materials, such as photoresist (model:SU-8) [1], poly(dimethyl)siloxane [2], polyimide [3], and poly-(para-chloro-xylylene) (Parylene C), have been investigated as packaging materials in many applications, including consumer electronics, lab-on-a-chip analysis, and prosthetic devices [4].

Three-dimensional (3D) printing has attracted considerable attention because it enables the fabrication of 3D structures with various shapes and diverse functions. Hull developed the first stereolithography (SLA) technology and patented the first 3D printer [5]. Current commercial 3D printers are based on various technologies, including liquid-based 3D printing technologies, such as SLA, digital light projection (DLP), inkjet printing, and PolyJet [6,7,8,9]; filament- or paste-based 3D printing technologies, such as fused deposition (FDM), 3D dispensing, robocasting, and laminated object manufacturing [10,11,12,13]; and powder-based 3D printing technologies, such as selective laser sintering, selective laser melting, electron beam melting, 3D powder binding, and laser engineered net shaping [14,15,16,17,18]. These technologies meet various types of user requirements. However, a need exists for the direct fabrication of biocompatible devices.

With advances in 3D printing, various 3D-printed microfluidic devices can now be used in different biomedical and biochemical applications, such as point-of-care diagnostics, cancer screening, and drug testing. In these applications, surface wettability has a crucial influence on microfluidics. For example, a study indicated that the contact angle of clear resin fabricated though SLA was 79° [19]. The contact angle of PolyJet material, such as Veroclear, was measured in [20]. The contact angles of glossy and matte surfaces were reported in the aforementioned study, and the contact angle exhibited a hydrophilic trend.

Droplets of various fluids may be stretched when they are transported inside microchannels; however, few studies have investigated the surface roughness of microchannels. A study indicated that surfaces printed through DLP and SLA are smoother than those printed through FDM [21]. However, the surface roughness of microfluidic chips printed through FDM can enhance fluid mixing [22]. The surface roughness of microchannels with various geometries that were fabricated through FDM was examined in [23], and the results indicated that higher water retention occurred in spiral microchannels than in linear and curved microchannels. Surface roughness may depend on the printing settings, such as the nozzle diameter, layer thickness, infill properties, and layer overlapping.

Parylene C has received considerable research attention because of its biocompatibility, chemical inertness, and low moisture permeability, which result in it having suitable barrier properties [24,25,26,27,28]. This material has been used in implantable medical devices, such as implantable nerve recording electrodes [29,30], implantable biomedical chips [31,32], drug delivery systems [32,33], spinal cord stimulators [34,35], and cardiac rhythm devices.

Although Parylene C has favorable barrier properties, it can exhibit moisture permeation [36]. Moreover, the effect of surface functionalization is crucial for understanding the wettability of Parylene C [37]. In a previous study, the wettability and surface energy of Parylene C were determined by measuring its water contact angle and using semiempirical theories, respectively [38]. Studies have developed printed parts for contact with biological samples [39]. The cytotoxic residues could have resulted from the cured resin. The formation of residues could be prevented by coating the printed parts with Parylene C. Furthermore, in [40], 3D-printed cell culture devices with Parylene C completely protected human mesenchymal stem cells from toxic effects. The wettability of Parylene C could affect cell adhesion and improve biocompatibility [41]. However, limited research has investigated the effects of solutions on Parylene C on 3D-printed photopolymer samples.

In the present study, the effects of 10% sodium hydroxide, 10% ammonium hydroxide, and 100% phosphate-buffered saline (PBS) on the contact angle and surface roughness of Parylene C on 3D-printed photopolymer samples were investigated. The adopted photopolymers were selected because of their increased use in soft biocompatible devices. Furthermore, the properties of 3D-printed photopolymers with Parylene C under weakly alkaline, strongly alkaline, and physiological saline environments were compared. The purpose of this study was to determine the effects of different solutions on the Parylene C coated on different 3D photopolymers. In this study, the contact angle and surface roughness of Parylene C were determined using a commercial contact angle measurement system and laser confocal microscope, respectively.

## 2. Materials and Methods

### 2.1. Materials

**Figure 1:**
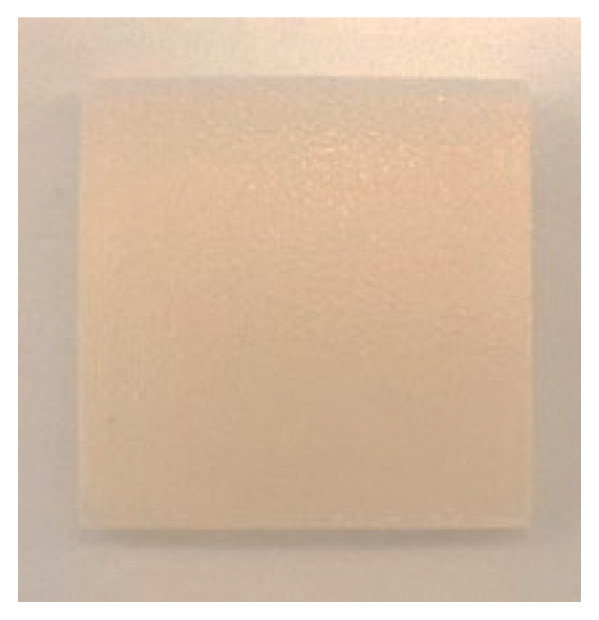
3D printed photopolymer substrate.

In this study, 3D-printed photopolymers were fabricated using a PolyJet 3D printer (J750, Stratasys, Eden Prairie, MN, USA). Figure 1 illustrates that the size of the substrates used in this study was 30 mm × 30 mm × 5 mm (thickness). These substrates comprised two photopolymers: VeroWhite 835 and Agilus 30. Photopolymers with different Shore A hardness values were obtained by adjusting the ratio of VeroWhite 835 and Agilus 30. The Shore A hardness 95 consists of 95% VeroWhite 835 and 5% Agilus 30. The Shore A hardness 85 consists of 85% VeroWhite 835 and 15% Agilus 30. The Shore A hardness 50 consists of 50% VeroWhite 835 and 50% Agilus 30.

### 2.2. Sample Preparation

The substrates were precleaned through an ultrasonic cleaning process by using deionized water. They were then air-dried and placed onto a substrate holder. Subsequently, Parylene C was deposited onto the substrates through CVD. The CVD process for growing Parylene C films involved the following steps: (a) vaporization of a dimer (di-para-xylylene) by the heating element of a Parylene C coating system (LH300, La Chi Enterprise, New Taipei City, Taiwan) at 150 °C and 133.32 Pa; (b) cracking of the dimer vapor into a monomer (para-xylylene) gas at 650 °C and 66.66 Pa; and (c) reaction of the monomer gas with the substrate, which resulted in the formation of a Parylene C film at 25 °C and 13.33 Pa. The overall thickness of the Parylene C film was set as 1.5 µm for all the substrates.

### 2.3. Sample Characterization

To conduct contact angle measurements, the Parylene C samples were precleaned through ultrasonic cleaning and then dried. All the contact angle measurements were performed according to the sessile drop method by using a commercial contact angle measurement system (FTA188, First Ten Angstroms, Newark, CA, USA). Distilled water was added on the sample surfaces, and the corresponding contact angles were recorded. The volume of the distilled water droplets in this study was 2 µL. Thirty substrates coated with Parylene C and exhibiting different Shore A hardness values were used for the contact angle and surface roughness measurements. Three measurements were conducted on each sample, and the mean value was calculated. Moreover, the measurements obtained for the samples when they were soaked in 10% sodium hydroxide, 10% ammonium hydroxide, and 100% PBS were compared. The soaking time in each solution was 2 h at a temperature of 25 °C and a humidity of 50%.

The arithmetic average surface roughness of the Parylene C samples was measured using a laser confocal microscope (VK-9710, Keyence, Japan). The vertical resolution of the adopted laser confocal microscope was 0.001 µm. The height of a point on the sample surface was measured using the encoder of the microscope. In this manner, surface profiles were obtained for the samples. The surface roughness of the samples was then derived on the basis of these profiles [42]. The surface roughness test was performed at three points. In addition, the surface morphology was determined using a desktop scanning/scanning transmission electron microscope (temic EM200S, Hsinchu, Taiwan).

## 3. Results

### 3.1. Effect of Solutions on the Contact Angle of Parylene C

**Figure 2:**
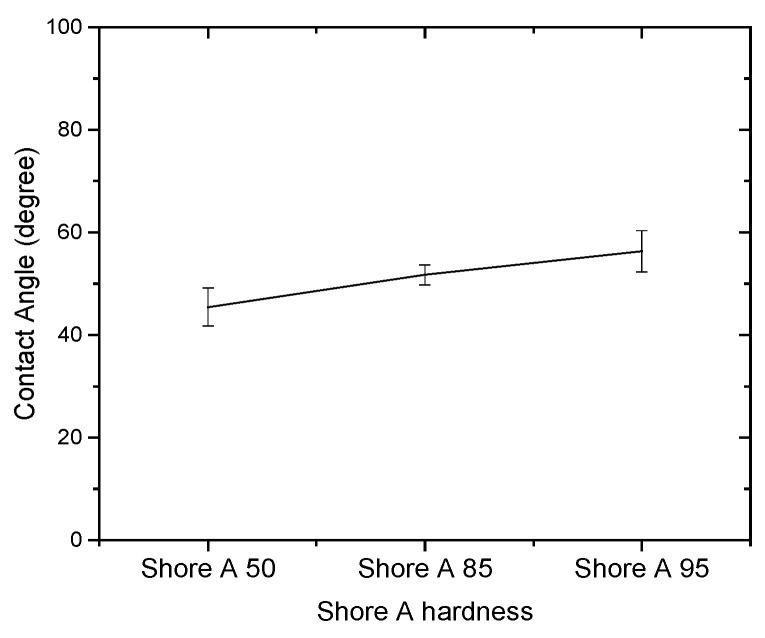
Contact angles of distilled water on substrates with different Shore A hardness values.

**Figure 3:**
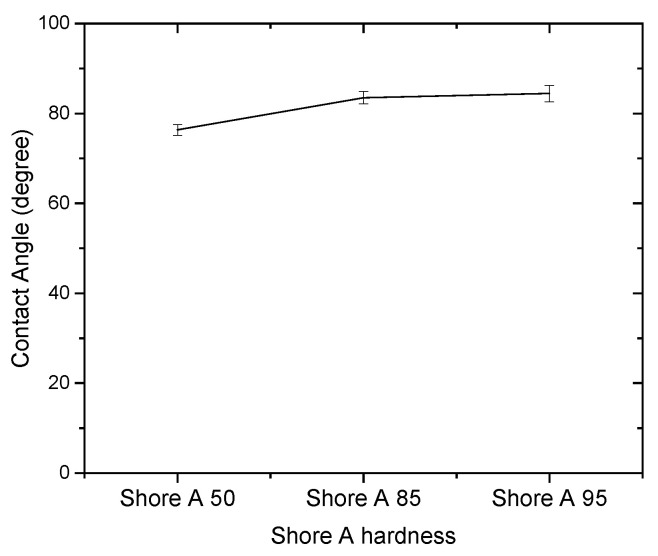
Contact angles of distilled water on Parylene C coated on substrates with different Shore A hardness values.

**Figure 4:**
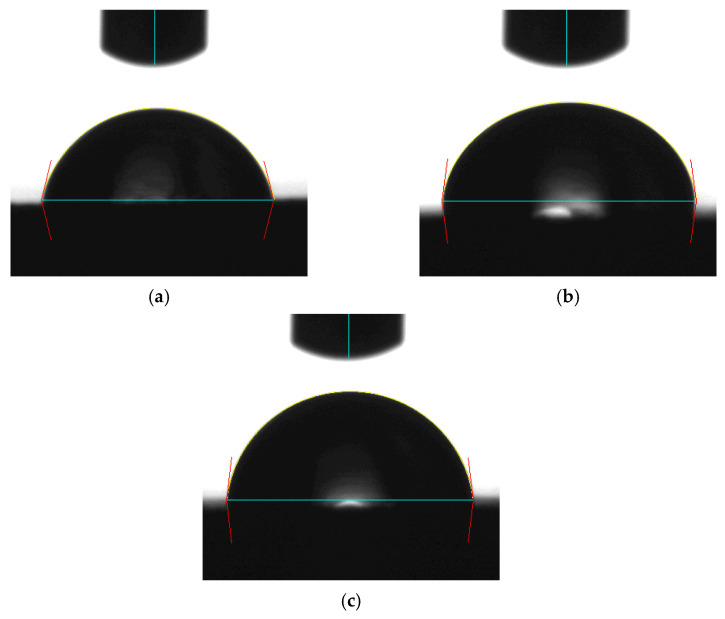
Typical contact angle images of distilled water on Parylene C coated on substrates with a Shore A hardness of (**a**) 50, (**b**) 85, and (**c**) 95.

**Figure 5:**
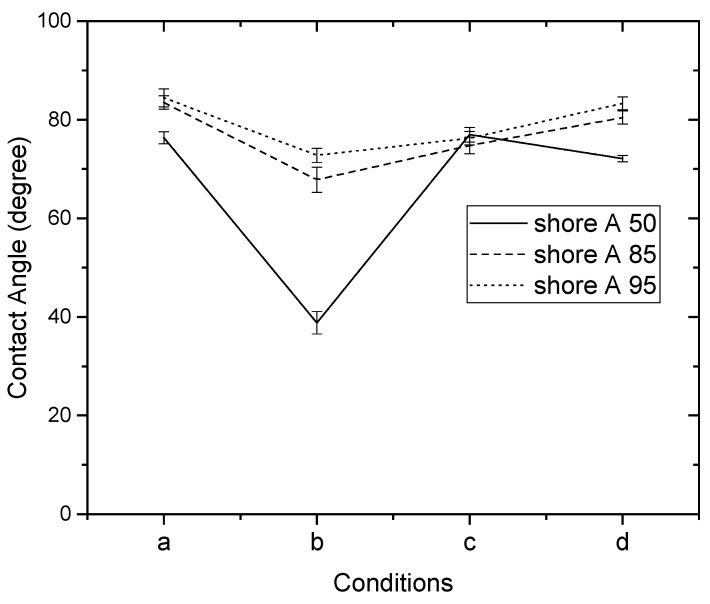
Contact angles of distilled water on Parylene C coated on substrates with different Shore A hardness values: (a) unsoaked, (b) soaked in 10% sodium hydroxide, (c) soaked in 10% ammonium hydroxide, and (d) soaked in 100% phosphate-buffered saline.

Since the contact angle hysteresis is critical [43], the receding and advancing contact angles were investigated experimentally. Table 1 shows the receding contact angles, advancing contact angles, and contact angle hysteresis for substrates without Parylene C. These mean values were obtained by averaging at least three independent measurements. The results revealed that there is a high mean contact angle hysteresis. The contact angles on the substrates without Parylene C are presented in Figure 2. The results observed in Figure 2 indicate that the contact angles of the substrates increased as Shore A hardness values increased. This suggests that the substrates with high contact angles are related to surface roughening. Next, the contact angles of Parylene C soaked in different solutions were compared. Figure 3 depicts the contact angles of Parylene C on substrates with three Shore A hardness values (50, 85, and 95) when the samples were not soaked in solutions. The contact angle of Parylene C on the substrate with a Shore A hardness of 50 was marginally lower than those on substrates with Shore A hardness values of 85 and 95. Although the observed contact angles were less than 90°, the aforementioned contact angles are quantitatively similar to those obtained by Tan et al. [44]. Tan et al. reported that the contact angle of Parylene C is 87° [44]. The contact angles of Parylene C on a silicon substrate were measured by Bi et al. [45]. The results are close to previously reported results [44]. The contact angle results indicated that a strong interaction occurred between Parylene C and distilled water. The contact angles on PDMS were measured and found to be 114.9° for DI water by Brancato et al. [46]. Figure 4 shows typical contact angle images of Parylene C on substrates with different Shore A hardness values. In this figure, the contact angle of Parylene C on the substrate with a Shore A hardness of 50 is flatter than those on the substrates with Shore A hardness values of 85 and 95. This result is consistent with the measurements depicted in Figure 3. The relatively flat contact angle of Parylene C on the substrate with a Shore A hardness of 50 may be related to the high energy of the substrate surface. Subsequently, the effects of solutions on the contact angle of Parylene C on the different substrates were compared. Figure 5 presents the contact angles of Parylene C on substrates with different Shore A hardness values when the substrates were soaked in 10% sodium hydroxide, 10% ammonium hydroxide, and 100% PBS. As displayed in Figure 5, soaking in 10% ammonium hydroxide did not appreciably change the contact angle of Parylene C on the substrate with a Shore A hardness of 50. However, the contact angle of Parylene C decreased when the substrates were soaked in 10% sodium hydroxide. This result indicates that 10% sodium hydroxide reacted with Parylene C. Moreover, all the solutions affected the contact angle of Parylene C on the substrate with a Shore A hardness of 85. As illustrated in Figure 5, 100% PBS had no major effect on the contact angle of Parylene C on the substrate, with a Shore A hardness of 95.

### 3.2. Effect of Solutions on the Surface Roughness of Parylene C

**Figure 6:**
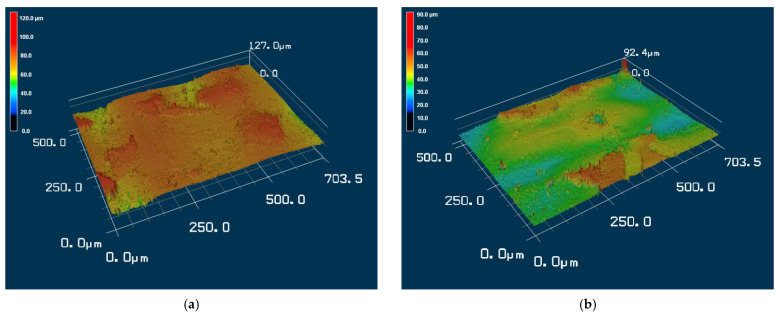
Three-dimensional topographies of the substrate with a Shore A hardness of 95: (**a**) without and (**b**) with Parylene C coating.

**Figure 7:**
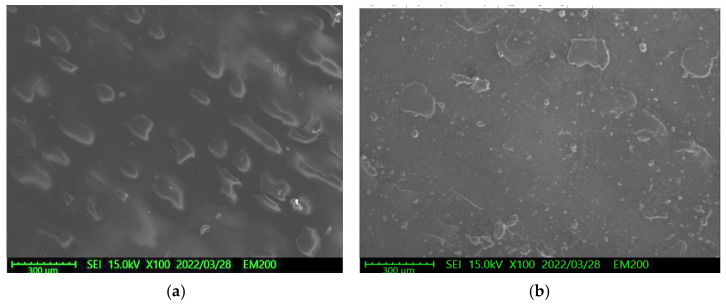
Surface SEM morphologies of the substrate with a Shore A hardness of 95: (**a**) without and (**b**) with Parylene C coating.

**Figure 8:**
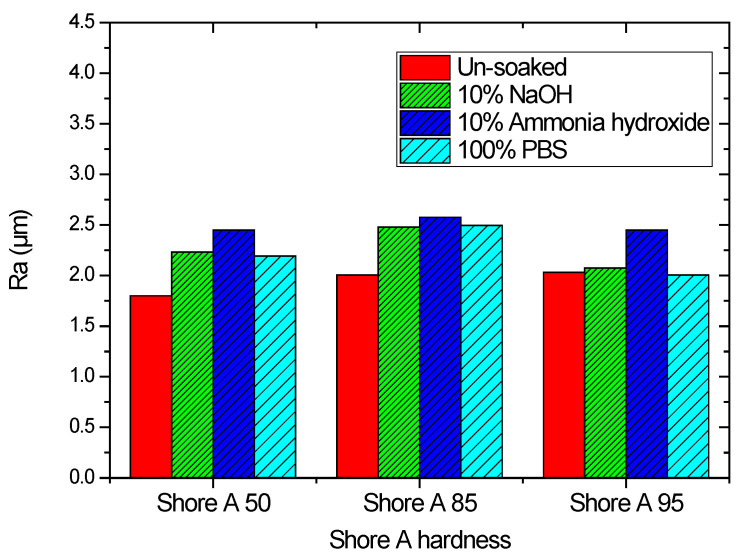
Arithmetic average roughness of substrates with different Shore A hardness values that were coated with Parylene C.

**Figure 9:**
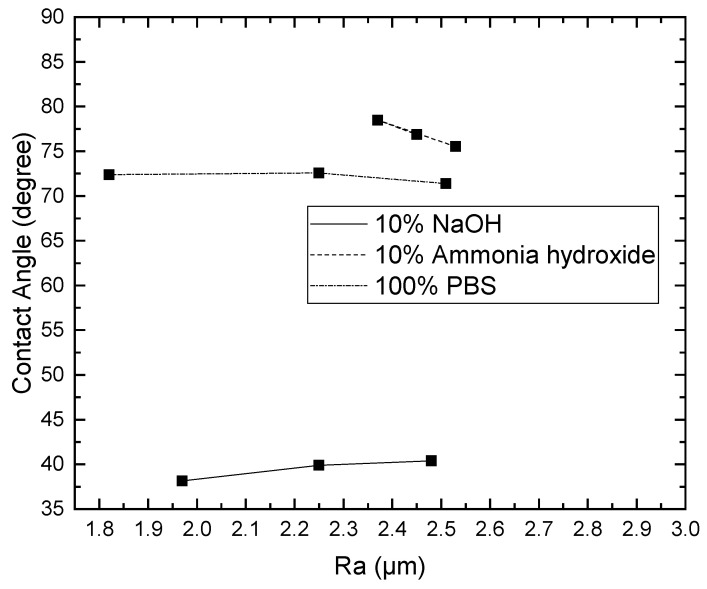
Contact angles of distilled water on Parylene C coated on a substrate with a Shore A hardness of 50 soaked in different solutions.

**Figure 10:**
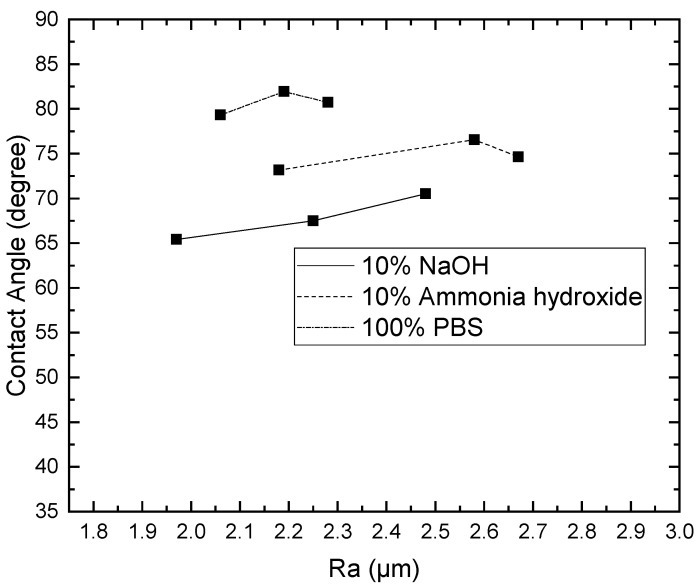
Contact angles of distilled water on Parylene C coated on a substrate with a Shore A hardness of 85 soaked in different solutions.

**Figure 11:**
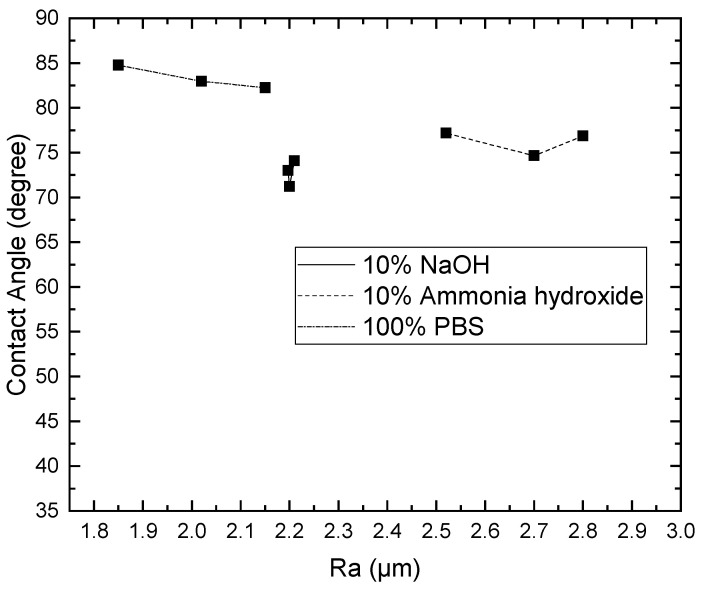
Contact angles of distilled water on Parylene C coated on a substrate with a Shore A hardness of 95 soaked in different solutions.

To examine the effects of the aforementioned three solutions on the surface roughness of the samples, the arithmetic average roughness (Ra) was experimentally investigated through the laser confocal microscope. In this paper, Ra is the arithmetic average of the ratio of the absolute values of the measured profile height deviations to the evaluation length. Table 2 presents the Ra values of the substrates with and without Parylene C. As demonstrated in Table 2, the Ra values of the substrates with Parylene C coating were lower than those of the uncoated substrates. This result is consistent with that obtained for a different substrate by Verwolf et al. [47]. Figure 6a,b shows the 3D topographies of the substrate with a Shore A hardness of 95 without and with Parylene C coating, respectively. These 3D topographies were the surface profiles extracted from measurement data of the laser confocal microscope. The results obtained in Figure 6 and Table 2 exhibit an identical trend. Figure 7a,b shows the surface area of the substrate with a Shore A hardness of 95 without and with Parylene C coating, respectively. The grains observed in Figure 7a are probably a result of the polymerization by UV exposure. In addition, some voids were observed when the substrate with a Shore A hardness of 95 coated with Parylene C (Figure 7b). Figure 8 depicts the Ra values of the substrates with different Shore A hardness values. The Ra value for the substrate with a Shore A hardness of 50 was smaller than those for the substrates with Shore A hardness values of 85 and 95. The Ra value increased with the Shore A hardness when the substrates were not soaked. Subsequently, the effects of the three solutions on the Ra values of the substrates coated with Parylene C were compared. The substrate with a Shore A hardness of 50, coated with Parylene C, exhibited a higher Ra value when soaked in 10% ammonium hydroxide than when soaked in the other two solutions. This result might be caused by the presence of neutral ammonia molecules in the solution. The Ra value was high when the substrate coated with Parylene C, with a Shore hardness of 85, was soaked in 10% ammonium hydroxide. This result is consistent with that obtained for the substrate with a Shore A hardness of 50. These results imply that the reaction between the alkaline ammonium hydroxide and Parylene C caused the aforementioned behavior for the substrate with a Shore A hardness of 85. Except for soaking in 10% ammonium hydroxide, the solutions had a slight effect on the Ra values of the substrate coated with Parylene C and had a Shore A hardness of 95. Finally, based on the present data in Figure 5 and Figure 8, the correlations between roughness and contact angle (CA) can be presented in Figure 9, Figure 10 and Figure 11. Figure 8 shows that the contact angles of Parylene C on a substrate with a Shore A hardness of 50 decreased with increasing Ra when the substrate was soaked in 10% sodium hydroxide and 100% PBS. However, soaking in 10% ammonium hydroxide did not appreciably change the contact angle of Parylene C on the substrate with a Shore A hardness of 50. One explanation for this is that the surface topography changed due to neutral ammonia molecules in the solution. Figure 10 reveals that the contact angles of Parylene C on a substrate with a Shore A hardness of 85 decreased with increasing Ra when the substrates were soaked in 10% sodium hydroxide, 10% ammonium hydroxide, and 100% PBS. These results revealed that decreasing wetting may be explained by the roughness. Figure 10 depicts the contact angles of Parylene C on a substrate with a Shore A hardness 95 was decreased with decreasing Ra when the substrates were soaked in 10% sodium hydroxide and 100% PBS. The Ra value was high when the substrate coated with Parylene C, with a Shore hardness of 95, was soaked in 10% ammonium hydroxide. Due to the surface topography not changing, the contact angle was high when the substrate coated with Parylene C, with a Shore hardness of 95, was soaked in 100% PBS. The correlations between roughness and contact angle (CA) can be expressed as:

(a) For the substrate with Shore A hardness 50 that was coated with Parylene C and soaked in 10% sodium hydroxide

$$\mathrm{CA}=-1.365Ra^{2}+6.325Ra+33.19\;\mathrm{for}\;1.97\;\leq Ra\leq \;2.48\;µm$$

(b) For the substrate with Shore A hardness 50 that was coated with Parylene C and soaked in 10% ammonium hydroxide

$$\mathrm{CA}=-2.27Ra^{2\;}+9.76Ra\;+68.03\;\mathrm{for}\;2.37\;\leq Ra\leq \;2.53\;µm$$

(c) For the substrate with Shore A hardness 50 that was coated with Parylene C and soaked in 100% PBS

$$\mathrm{CA}=-0.38Ra^{2}\;+0.93Ra\;+72.03\;\mathrm{for}\;1.82\;\leq Ra\leq \;2.51\;µm$$

(d) For the substrate with Shore A hardness 85 that was coated with Parylene C and soaked in 10% sodium hydroxide

$$\mathrm{CA}=0.465Ra^{2\;}+0.685Ra+64.27\;\mathrm{for}\;2.37\;\leq Ra\leq \;2.48\;µm$$

(e) For the substrate with Shore A hardness 85 that was coated with Parylene C and soaked in 10% ammonium hydroxide

$$\mathrm{CA}=0.195Ra^{2}+0.925Ra+72.03\;\mathrm{for}\;2.18\;\leq Ra\leq \;2.67\;µm$$

(f) For the substrate with Shore A hardness 85 that was coated with Parylene C and soaked in 100% PBS

$$\mathrm{CA}=-1.91Ra^{2}+8.33Ra+72.91\;\mathrm{for}\;2.06\;\leq Ra\leq \;2.28\;µm$$

(g) For the substrate with Shore A hardness 95 that was coated with Parylene C and soaked in 10% sodium hydroxide

$$\mathrm{CA}=-0.355Ra^{2}+2.845Ra+68.75\;\mathrm{for}\;2.197\;\leq Ra\leq \;2.21\;µm$$

(h) For the substrate with Shore A hardness 95 that was coated with Parylene C and soaked in 10% ammonium hydroxide

$$\mathrm{CA}=2.36Ra^{2}-9.29Ra+83.81\;\mathrm{for}\;2.52\leq Ra\leq 2.8\;µm$$

(i) For the substrate with Shore A hardness 95 that was coated with Parylene C and soaked in 100% PBS

$$\mathrm{CA}=-2.16Ra^{2}+9Ra+75.4\;\mathrm{for}\;1.85\;\leq Ra\leq \;2.15\;µm$$

## 4. Discussion

This research investigated the effects of 10% sodium hydroxide, 10% ammonium hydroxide, and 100% phos-phate-buffered saline (PBS) on the contact angle and surface roughness of Parylene C on 3D-printed photopolymer samples. The findings of this study indicate that the contact angle of the sample with a low Shore A hardness was flatter than that of the samples with high Shore A hardness values. The contact angles of Parylene C on three substrates with different Shore A hardness values did not vary considerably when the substrates were soaked in 10% ammonium hydroxide. Moreover, the Ra values of the substrates with Parylene C coatings were lower than those of the uncoated substrates. Parylene C exhibited a high Ra value when the substrate was soaked in 10% ammonium hydroxide. The contact angles obtained for the unsoaked samples are in agreement with the results obtained by Tan et al. [44]. Furthermore, although the substrates used in this study and [45] are different, the Ra values of the substrates with Parylene C obtained in this study are in agreement with those proposed by Verwolf et al. [47].

The effects of solutions on the wettability and surface roughness of Parylene C coated on 3D-printed photopolymer samples were demonstrated. Our results provide an understanding of the effects of 10% sodium hydroxide, 10% ammonium hydroxide, and 100% PBS on the contact angle and surface roughness of Parylene C coated on substrates with different Shore A hardness values. It seems that substrates with different Shore A hardness values and Ra may have influenced to form Parylene C. Therefore, NNwe further speculated that the effects of solutions might affect the Ra of Parylene C coated on 3D-printed photopolymer samples.

Future research could explore the effect of the print settings on the substrates. For example, the surface roughness of Parylene C may depend on the print settings, such as the nozzle diameter, layer thickness, infill properties, and layer overlapping. In addition, microfluidic devices fabricated through 3D printing may contain cytotoxic residues when the cured resin is used for printing. The formation of cytotoxic residues can be prevented by coating such devices with Parylene C. Moreover, Bae and Lee found that the wettability and interface of Parylene C have crucial influences on its practical use [37]. The water vapor transport data of Parylene C were conducted by Hubbel et al. [48]. They found that the Parylene C has the lowest diffusion and solubility coefficient compared with Mylar A and Kapton H. Parylene C is also recognized for its low water vapor transport rate [49]. However, this has not been investigated in our study. The novelty of our study presented here provides empirical relation between roughness and contact angle for Parylene C on various Shore hardness of 3D-printer photopolymer substrates for future studies to assess the wetting characteristics.

## 5. Conclusions

This study investigated the effects of solutions on the contact angle and surface roughness of Parylene C coated on 3D-printed photopolymer substrates. The obtained contact angle data indicated that 10% ammonium hydroxide had no major effect on the contact angle of Parylene C on a substrate with a Shore A hardness of 50. However, 10% sodium hydroxide, 10% ammonium hydroxide, and 100% PBS considerably affected the contact angle of Parylene C on a substrate with a Shore A hardness of 85. Among the aforementioned three solutions, 100% PBS exhibited no major effect on the contact angle of Parylene C on a substrate with a Shore A hardness of 95. In addition, the Ra values of samples with Parylene C coating were lower than those of uncoated samples. Samples coated with Parylene C exhibited high Ra values when soaked in 10% ammonium hydroxide. Finally, the correlations between roughness and contact angle were proposed. The results of this study indicate the potential for the application of polymer-based biocompatible devices.

## Figures and Tables

**Table 1:** Contact angle results of substrates without Parylene C coating.

Substrate Material	Mean Receding Contact Angle (Degree)	Mean Advancing Contact Angle (Degree)	Mean Contact Angle Hysteresis (Degree)
Shore A hardness 50	43.79	47.09	3.30
Shore A hardness 85	50.20	53.26	3.06
Shore A hardness 95	56.14	62.52	6.38

**Table 2:** Arithmetic average surface roughness (Ra) of uncoated and coated substrates.

Substrate Material	Ra (µm)	
	Uncoated Parylene C	Coated Parylene C
Shore A hardness 50	2.48	1.8
Shore A hardness 85	2.91	2.01
Shore A hardness 95	3.06	2.32

## Data Availability

Not applicable.
